# Concordance between somatic copy number loss and down-regulated expression: A pan-cancer study of cancer predisposition genes

**DOI:** 10.1038/srep37358

**Published:** 2016-12-08

**Authors:** Ran Wei, Ming Zhao, Chun-Hou Zheng, Min Zhao, Junfeng Xia

**Affiliations:** 1 Institute of Health Sciences, School of Computer Science and Technology, Anhui University, Hefei, Anhui 230601, China; 2 School of Engineering, Faculty of Science, Health, Education and Engineering, University of Sunshine Coast, Maroochydore DC, Queensland, 4558, Australia

## Abstract

Cancer predisposition genes (CPGs) are a class of cancer genes in which germline variants lead to increased risk of cancer. Research has revealed that copy number variation (CNV) may be linked to cancer susceptibility in CPGs. In this pan-cancer analysis, we explored the relationship between somatic CNV and gene expression changes in CPGs. Based on curated 827 human CPGs from literature, we firstly identified 729 CPGs with precise CNV information from 5067 tumor samples using TCGA CNV data. Among them, 128 CPGs tended to have more frequent copy number losses (CNLs) compared with copy number gains (CNGs). Then by correlating these CNV data with TCGA gene expression data, we obtained 49 CPGs with concordant CNLs and gene down-regulation. Intriguingly, five CPGs showed concordance between CNL and down-regulation in 50 or more tumor samples: *MTAP* (216 samples), *PTEN* (143), *MCPH1* (86), *SMAD4* (63), and *MINPP1* (51), which may represent the recurrent driving force for gene expression change during oncogenesis. Moreover, network analysis revealed that these 49 CPGs were tightly connected. In summary, this study provides the first observation of concordance between CNLs and down-regulation of CPGs in pan-cancer, which may help better understand the CPG biology in tumorigenesis and cancer progression.

The genetic basis of inherited predisposition to cancer has been recognized through observation of unusual clustering of cancer within families and between twins. It is estimated that approximately 3% of cancers result from an inherited susceptibility^1^. Over 100 cancer predisposition genes (CPGs) have been discovered using candidate gene approach and high-throughput strategies like genome-wide mutation analysis^1^. Identification and understanding of CPGs will not only help us to explore the biological pathways involved in cancer predisposition but also provide information that can be used to reduce the likelihood of developing cancer in healthy individuals and optimize the management of individuals with cancer.

To explore the molecular mechanism of cancer susceptibility at a systems-biology level, we have developed a literature-based gene resource for CPGs^2^. In total, 827 human CPGs were collected from published articles. We also found there is an overlap between CPGs and somatically mutated cancer genes. According to ‘two-hit’ hypothesis, carcinogenesis is a consequence of the accumulation of germline and somatic mutations^3^. Therefore, it is important to integrate germline and somatic data to identify important genes and molecular pathways involved in cancer biology^4^,^5^,^6^,^7^.

In the present study, using somatic copy number variation (CNV) and gene expression data from The Cancer Genome Atlas (TCGA) and the human CPGs with germline variants from dbCPG, we aimed to investigate the systematic relationship of somatic CNVs and gene expression in cancer predisposition. In order to present a global view of the recurrent somatic CNVs across multiple cancer types, we conducted a pan-cancer based study instead of a single specific cancer type in human CPGs.

## Results

### Biological features of CPGs with frequent copy number loss in multiple cancer types

**Figure 1:**
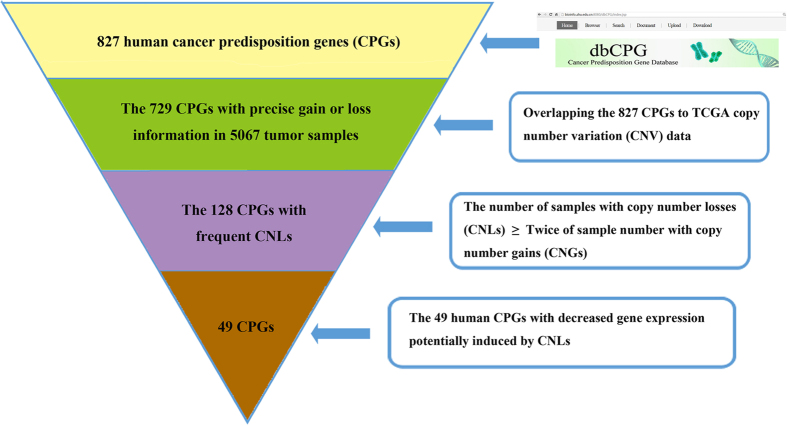
The work flow for identifying 49 CPGs with concordance between CNLs and down-regulation.

To investigate somatic CNVs information in CPGs, we downloaded 827 human CPGs (724 protein-coding, 23 non-coding and 80 unknown type genes (the type of gene is labelled as ‘unknown type’ in NCBI)) from dbCPG, a database focused on human cancer predisposition genes based on literature^2^. Utilizing the University of California Santa Cruz (UCSC) Table Browser^8^, we obtained precise genomic locations of these 827 CPGs in GRCH38. Then, we intersected all the 827 human CPGs to all TCGA CNVs data derived from COSMIC^9^. In total, 729 human CPGs overlapped with at least one CNVs according to their genomic coordinates. We further deleted non-informative CNVs due to the lack of matched control tissue. Since most of CPGs act as tumor suppressors that play their roles by loss of function^1^, we set a threshold of 2 to identify those frequent CNLs events. In other words, we only included those CPGs which the number of samples with CNLs was at least twice that of the number of samples with CNGs (Fig. 1) for the following analysis. In total, we harvested 128 human CPGs to further investigate functional enrichment analysis and integrative gene expression analysis (Supplementary Table S1).

**Figure 2:**
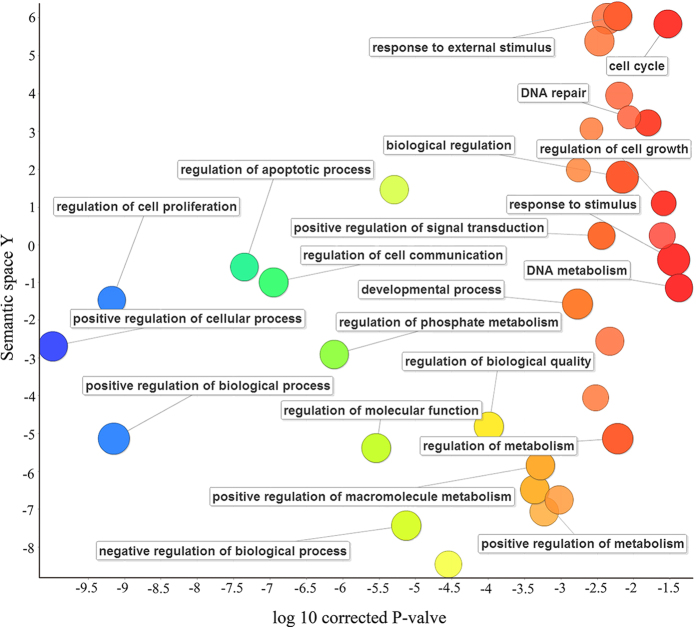
Gene Ontology (GO) analysis of 128 human CPGs with frequent CNLs. The scatter plot shows the GO clusters for the 128 CPGs in a two dimensional space derived by applying multidimensional scaling to a matrix of GO terms’ semantic similarities. Bubble colors represent the log of corrected P-value (bubbles with deep colors corrected P-value are larger); bubble sizes indicate the frequency of the GO term in the GOA database (bubbles with more general terms are lager).

To understand the biological features, we performed the gene ontology (GO) enrichment analysis on 128 human CPGs with frequent CNLs by using the DAVID server^10^. We collected the enriched GO terms with an adjusted P-value < 0.05 as calculated using a hypergeometric test followed by the Benjamini-Hochberg correction (Supplementary Table S2). We used the online tool REVIGO^11^ to integrate non-redundant GO terms and visualize those significant GO terms based on semantic similarity (Fig. 2). The majority of terms are related to cell cycle, growth, proliferation, and apoptosis, all of which play an important part in the occurrence and development of cancer cells. The 128 human CPGs with CNLs also participate in fundamental biological process, such as regulation of cell communication, cell metabolism and other cellular process. To confirm whether these pathways are specific to the 128 CPGs with CNL, we calculated the frequency of each GO term using 100 permutations of the randomly generated 128 CPGs from 729 CPGs with precise CNV information utilizing DAVID^10^. The result shows that several pathways are indeed specific to those CPGs with CNL, including ‘activation of protein kinase activity’ (GO: 0032147), ‘regulation of cell-matrix adhesion’ (GO: 0001952), and ‘regulation of cell-substrate adhesion’ (GO: 0010810) (Supplementary Table S3). These three processes are related to cell communication or metabolism, and may have an effect on carcinogenesis^12^,^13^.

### CPGs with concordant CNL and decreased gene expression are enriched

After consolidating the gene expression change of the TCGA samples with CNL for human CPGs, we investigated the correlation between CNLs and down-regulation for each CPG (Fig. 1). We used a threshold of Z-score that is same as COSMIC TCGA gene expression criteria to examine the gene expression level change of those CPGs in a specific TCGA sample. Specifically, we utilized a Z-score of below −2 to identify decreased expression CPGs in a specific TCGA sample.

**Figure 3:**
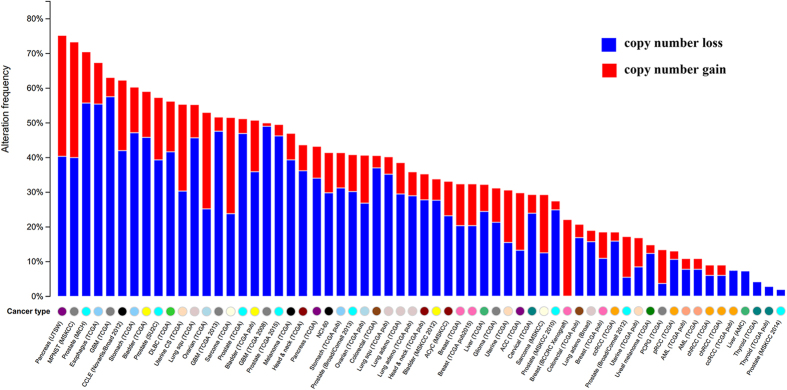
A pan-cancer global view of CNV features based on 49 CPGs with decreased gene expression induced by CNLs.

By examining the TCGA tumor samples with both expression and CNV information, we obtained 49 human CPGs with decreased gene expression levels that were associated with a loss of gene copy number in the same tumor sample (Supplementary Table S4). To explore the 49 CPGs with concordance between CNLs and down-regulation, we analyzed the CPGs CNV mutational patterns in pan-cancer utilizing cBioPortal^14^ (Fig. 3). In the pancreatic cancer cohort (UTSW, the University of Texas Southwestern Medical Center), there were 82 cases (75.20%) that had at least one CPG with CNV (Supplementary Table S5). We found approximately 40% of pancreatic cancer patients have at least one CNL in one of the 49 CPGs. Similarly, in malignant peripheral nerve sheath tumor (MSKCC, Memorial Sloan-kettering Cancer Center), 11 cases (73.30%) had copy number change and about 40% patients had at least one CNL. Greater than 50% of copy number alterations is found in CCLE and other cancer types, including metastatic prostate cancer, esophageal carcinoma, glioblastoma, stomach adenocarcinoma, bladder urothelial carcinoma, lymphoid neoplasm diffuse large B-cell lymphoma, uterine carcinosarcoma, lung squamous cell carcinoma, ovarian serous cystadenocarcinoma, and sarcoma. Furthermore, there were three cancer types (metastatic prostate adenocarcinoma, esophageal carcinoma, and glioblastoma) that contained over 50% patients with CNLs (Fig. 3). We also found the majority of patients in these cancer cohorts have more CNLs comparison with CNGs. Overall, The CNVs information in multiple cancers and cell lines may illustrate that the 49 human CPGs with concordance between CNLs and down-regulation, play a critical role in cancer development via massive CNLs.

To assess the biological features of 49 human CPGs with concordance between CNLs and down-regulation, we also performed the enrichment analysis by utilizing DAVID^10^. We collected the GO terms with an adjusted P-value less than 0.05 as calculated using a hypergeometric test followed by the Benjamini-Hochberg correction (Supplementary Table S6). Not surprisingly, these 49 human CPGs are overrepsented in regulation of apoptosis and programmed cell death, which highlight their fundamental roles on controlling cell growth.

**Figure 4:**
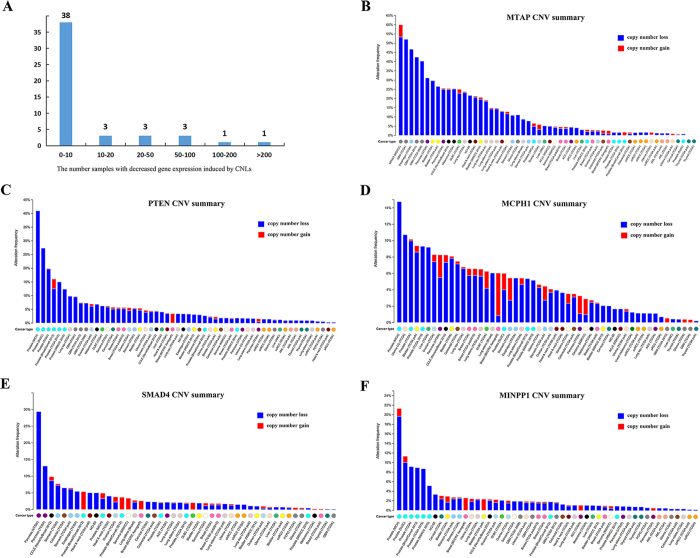
The number of genes with decreased gene expression induced by CNLs and a pan-cancer view of CNV distribution in five CPGs: *MTAP, PTEN, MCPH1, SMAD4*, and *MINPP1*. (**A**) The number of CPGs with decreased gene expression induced by CNLs in different tumor samples. The CNV landscape for (**B**) *MTAP*; (**C**) *PTEN*; (**D**) *MCPH1*; (**E**) *SMAD4*; and (**F**) *MINPP1*.

To further investigate the gene expression change induced by CNLs, we counted the number of samples for all of the 49 CPGs. Interestingly, five genes with concordance between CNLs and down-regulation were observed in more than 50 tumor samples, including *MTAP* (216 samples), *PTEN* (143 samples), *MCPH1* (86 samples), *SMAD4* (63 samples), and *MINPP1* (51 samples) (Fig. 4A). We analyzed the CNV distribution in these five CPGs (Fig. 4B–F). *MTAP* had CNLs in approximately 60% cases in malignant peripheral nerve sheath tumor cohort from MSKCC (Fig. 4B). *PTEN* was altered in about 40% patients with prostate cancer from MICH dataset (Fig. 4C). Similarly, *MCPH1* and *MINPP1* exhibited frequent CNLs in the same prostate cancer cohort with *PTEN* (Fig. 4D,F). *SMAD4* was shown gene copy number loss in nearly 30% of cases in pancreatic cancer from UTSW (Fig. 4E). In summary, CNLs of these CPGs are showed different pattern in different cancer types.

**Figure 5:**
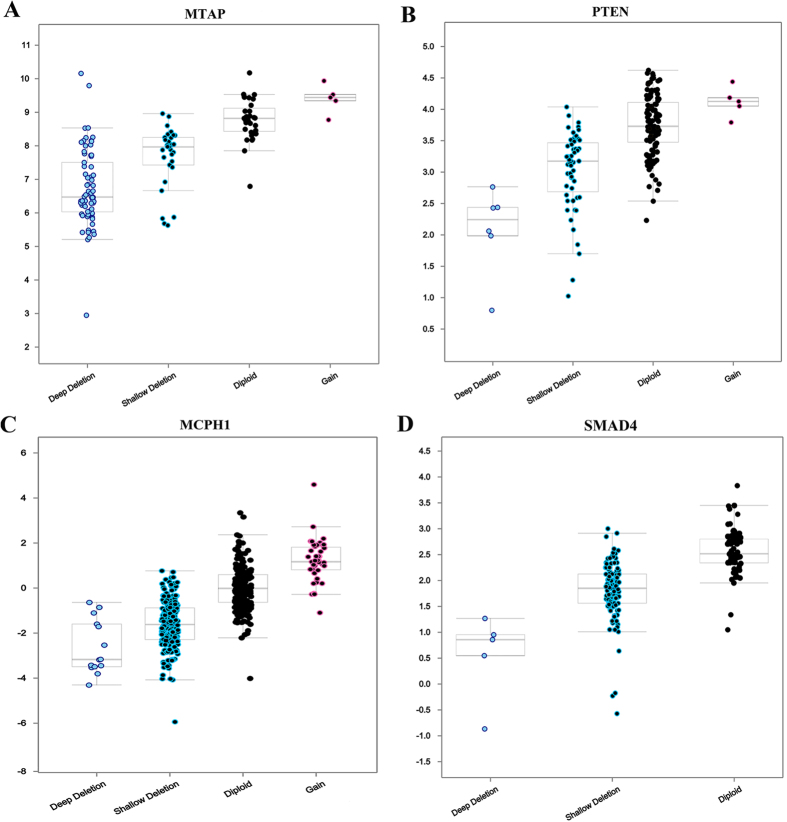
The correlation of CNVs and gene expression in four CPGs: *MTAP, PTEN, MCPH1*, and *SMAD4.* (**A**) *MTAP* using TCGA glioblastoma data; (**B**) *PTEN* using TCGA lung squamous carcinoma data; (**C**) *MCPH1* using TCGA breast cancer data; and (**D**) *SMAD4* using TCGA colorectal cancer data.

**Figure 6:**
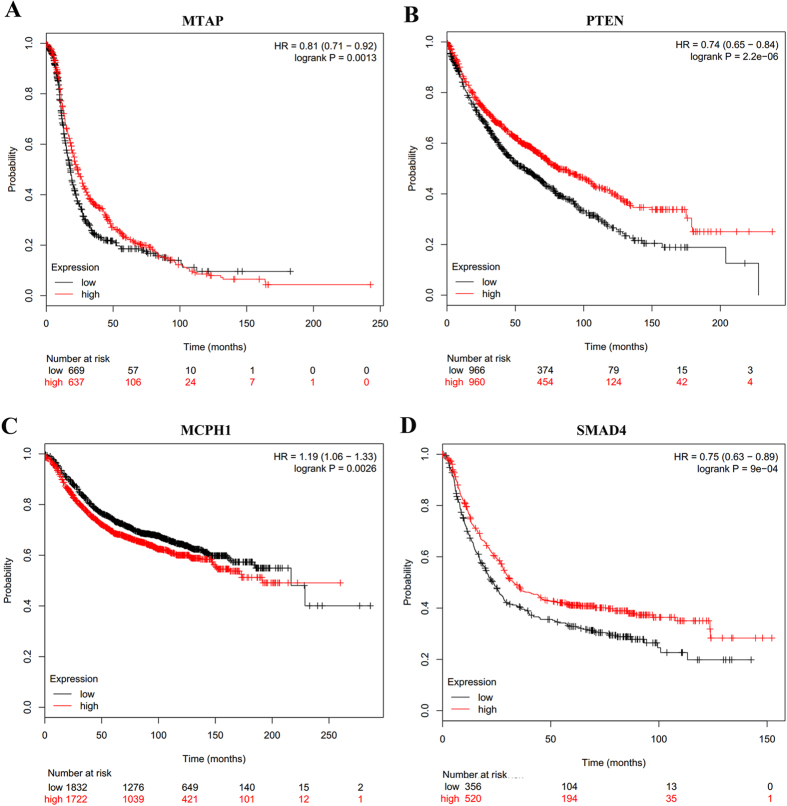
Survival analysis in four CPGs: *MTAP, PTEN, MCPH1*, and *SMAD4.* (**A**) *MTAP* using ovarian cancer data; (**B**) *PTEN* using lung cancer data; (**C**) *MCPH1* using breast cancer data; and (**D**) *SMAD4* using gastric cancer data.

To explain the relationship between gene expression changes and CNLs, we further concentrated on the five CPGs with the number of samples greater than 50. We used the data from five different cancers: TCGA glioblastoma, lung squamous carcinoma, breast cancer, colorectal cancer, and thyroid carcinoma. The deeper the deletion, the lower the gene expression of those CPGs in the tumor samples (Fig. 5). These results confirmed that gene expression decrease can be correlated with CNLs. To examine the potential diagnosis significance, we also conducted a survival analysis on four CPGs (*MTAP, PTEN, MCPH1*, and *SMAD4*) by using Kaplan-Meier plotter^15^ on different cancers. As shown in Fig. 6, we concluded that these 4 CPGs were significantly associated with the risk of corresponding most mutated cancer types, especially *PTEN* (HR = 0.74, P = 2.2e-06) and *SMAD4* (HR = 0.75, P = 9e-04). This observation further indicates the potential CNL-induced gene down-regulation may be associated with patient survival.

### A connected biological map of CPGs with decreased gene expression induced by CNLs

**Figure 7:**
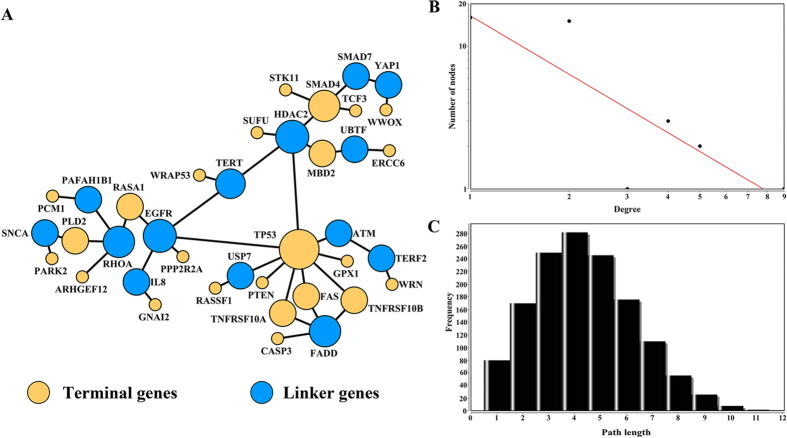
Reconstructed interaction sub-network for the 49 CPGs with decreased gene expression induced by CNLs. (**A**) The genes in yellow are terminal genes from the 49 CPGs with decreased expression induced by CNLs, and the other genes in blue represent linker genes that bridged the 46 terminal genes. The node size reflects the number of degree. A bigger size means more connection; (**B**) The degree distribution of all nodes (genes) in the network; (**C**) The distribution of the shortest path length.

To explore the common functions and better understand the cellular events associate with 49 human CPGs with decreased expression induced by CNLs, we performed a protein-protein interaction (PPI) analysis using the pathway annotation data from Pathway Commons database^16^. These interactions are built on the known biological pathways, including the Reactome pathway database and KEGG, and are useful for pathway reconstruction because they may avoid the high levels of noise, sparseness, and highly skewed degree distribution that is often observed in physical interaction-based PPI networks. To achieve the aim, we first mapped the 49 human CPGs to PPI network. Then, a sub-network was extracted to connect as many of the 49 CPGs as possible by utilizing Klein-Ravi algorithm^17^. The sub-network module contained 38 genes with 40 links (Fig. 7A). For 38 nodes, 24 are from the 49 CPGs with decreased gene expression caused by CNLs. The remaining 14 are linker genes bridging those CPGs, including *ATM, PAFAH1B1, YAP1, SNCA, TERT, RHOA, UBTF, IL8, HDAC2, FADD, TERF2, EGFR, USP7*, and *SMAD7*. Intriguingly, we found four linker genes are also CPGs, including *ATM, TERT, EGFR*, and *SMAD7*. These genes are not used as terminal genes (the known 49 CPGs in this study) because they did not show the concordant gene down-regulation and CNLs.

To further describe the sub-network, we analyzed the topological properties of those genes that are correlated with each other. The degree represents the number of edges adjacent to a node, which is one of the most noteworthy features in the PPI network. The degrees of the nodes in the reconstructed sub-network follows a power law distribution 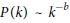, where 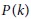 is the probability that one gene has a connection with other genes (*k*), and *b* is an exponent with an estimated value of 1.364 (Fig. 7B). Therefore the resulted network is different from other human PPI networks in which the majority of genes are connected with *b* exponent of 2.9^18^. Moreover, most of genes in the sub-network can be linked by three to five steps (Fig. 7C). Analyzing these topological features (degree and short path) and the results by using GeneRev^17^, we observed that most of CPGs in the sub-network tend to have a higher modularity^19^ (0.59), compared to randomly generated 49 CPGs from the full list of 729 CPGs with CNV information (0.52) or 128 CPGs with frequent CNLs (0.55) using 100 permutations, which indicates that the sub-network derived from 49 CPGs with concordance between CNL and down-regulation have dense connections. Acting in accordance with the tight connection, the hub nodes in the network may play important roles in regulating the biological process in cellular systems. In this sub-network, there are six nodes with at least four connections including *TP53* (9 connections), *HDAC2* (5), *EGFR* (5), *SMAD4* (4), *FADD* (4), and *RHOA* (4). It is not surprising that *TP53* is the hub node with most connections in the sub-network. As a common tumor suppressor gene, the mutation of *TP53* can increase the risk in diverse types of human cancer^20^. The *HDAC2* and *EGFR* are also consistent with the center of the sub-network with five connections. *HDAC2* (histone deacetylase 2) is a protein coding gene that codes a member of histone deacetylase family and plays an important role in cell cycle progression and transcriptional regulation, and has been linked with gastric cancer^21^. Epidermal growth factor receptor (*EGFR*), encoded a cell surface protein that binds to epidermal growth factor, play a significant role in lung cancer^22^. In addition, the remaining genes of *SMAD4, FADD*, and *RHOA* are associated with colorectal cancer^23^, breast cancer^24^, and testicular cancer^25^, respectively. In conclusion, we can obtain the relationships between those 49 human CPGs with concordance between CNLs and down-regulation and discover novel genes that may have similar mechanism for CNL-induced CPGs down-regulation.

## Discussion

Tumorigenesis is a complex interplay between germline susceptibility and somatic mutation^4^,^5^,^7^. CPGs are the genes that can highly or moderately increase the risk of cancer as a consequence of germline variant. The principal aim of this study is to better understand the relationship between CNV and gene expression change in human CPGs. We found that some CPGs exhibit concordance between CNLs and down-regulation in multiple cancer types, which consists with previous studies^1^. These CPGs with frequent CNLs participate in fundamental biological process and play a significant role in cancer-relation pathways. The CNLs in CPGs may contribute to gene expression change involving cancer development. In conclusion, our systematic analysis of the relationship between CNV and gene expression in human CPGs provide the first observation of concordance between CNLs and down-regulation of CPGs in tumor samples from different cancer types, which may help better understand the CPG biology in tumorigenesis and cancer progression.

## Materials and Methods

### The curated CPGs from thousands of literatures

To conduct a systematic CNV survey of CPGs, we downloaded 827 curated human CPGs (724 protein-coding, 23 non-coding and 80 unknown type genes (the type of gene is labelled as ‘unknown type’ in NCBI)) from dbCPG database^2^ in a plain text format with all gene ID and official symbol (http://bioinfo.ahu.edu.cn:8080/dbCPG/index.jsp). To intersect CPGs with CNVs, we first annotated these 827 CPGs with precise genomic locations. To achieve this we downloaded the corresponding genomic location data from the University of California Santa Cruz (UCSC) Table Browser (https://genome.ucsc.edu/index.html)^8^ and converted the RefSeq ID to gene official symbol by using the online tool BioMart^26^. Finally, an in-house shell script was implemented to extract the genomic coordinates of the 827 CPGs in GRCH38.

### Classification of CNV based on the TCGA pan-cancer data

To collect the CNVs with precise gain or loss information, we downloaded the TCGA CNV data with the GRCH38 genomic coordinates from Catalogue of Somatic Mutations in Cancer database (COSMIC) (V74). When integrating TCGA CNV data, we adopted COSMIC criteria to define the copy number loss (CNL) and copy number gain (CNG). CNL was obtained using the following criteria: (the average genome ploidy < = 2.7 AND total DNA segment copy number = 0) OR (average genome ploidy > 2.7 AND total DNA segment copy number < (average genome ploidy −2.7)). Similarly, criteria for CNG were as follows: (the average genome ploidy < = 2.7 AND total DNA segment copy number > = 5) OR (average genome ploidy > 2.7 AND total DNA segment copy number > = 9). We then intersected all the CNV regions with CPGs with BEDTools^27^. By overlapping all the CNG and CNL information to all the 827 CPGs with GRCH38 coordinates, we obtained 729 CPGs with precise gain or loss information. To provide cross-validation, we calculated the number of samples with CNG or CNL regardless of cancer type. Based on previous work by Rahman^1^ and our analysis, the majority of CPGs act as tumor suppressors with mutations that cause loss-of-function in cancer progression. Consequently, we collected those CPGs with more CNLs than CNGs. Specifically, a cut-off of 2 was set to collect those CPGs having at least twice of tumor samples with CNLs as tumor samples with CNGs. As a result, we obtained 128 CPGs with more CNLs than CNGs for the following integrative gene expression analysis.

Furthermore, to reveal the biological process and cellular function of those 128 CPGs with frequent CNLs, we performed the Gene Ontology (GO) enrichment analysis by using DAVID^10^. A web server, REVIGO^11^, was utilized to summarize long lists of GO terms by finding a representative subset of the terms using a clustering algorithm that was based on semantic similarity measures.

### Gene expression analysis for CPGs with CNLs

To investigate the gene expression changes caused by CNV for CPGs, we downloaded the TCGA gene expression data from the COSMIC database (V74). In this study, we focused only on those gene expression changes in matched TCGA samples with CPG CNLs. The RSEM quantification results from the RNAseq V2 platforms in COSMIC were used to indicate the accurate transcript quantification of RNA-Seq data. The average and sample standard deviation of gene expression values were calculated based on those tumor samples that were diploid for each corresponding gene.

The standard Z-score was used to characterize whether a CPG is over or under expressed. A threshold of Z-score with absolute value 2 was used. The Z-score over 2 was defined as the increased gene expression while the Z-score less than −2 represented under expression. For those 49 CPGs with concordance between CNLs and down-regulation, we further systematically analyzed their CNV patterns in pan-cancer of TCGA samples using cBioPortal^14^. Furthermore, to assess the function of 49 CPGs with CNL-associated gene expression change, we also explored the functional enrichment analysis using DAVID^10^ with adjusted P-value less than 0.05 (Supplementary Table S6).

### Sub-network extraction for the CPGs with concordance between CNLs and down-regulation

To investigate the interaction features of those CPGs with frequent CNLs and consistent gene down-regulation, we extracted a sub-network to connect 49 CPGs with the rest human genes. First, we constructed the protein-protein interaction (PPI) network derived from the Pathway Commons database^16^, which contained 3,629 proteins and 36,034 PPIs. It is worth noting that this integrated human interaction data is based on well-curated pathway database (HumanCyc, Reactome, and KEGG pathway database^28^). Thus, the interactions we used have biological meaning rather than physical interactions. Based on these pathway-based interactions, we used Klein-Ravi algorithm in GeneRev^17^ to extract the sub-network related to 49 CPGs. In this sub-network extraction strategy, all of the 49 CPGs were used as seed genes to map onto the pathway-based interactions. A sub-network with as many seed CPGs as possible was formed by connecting the involved CPGs through their shortest paths. Finally, we used Cytoscape (V2.8.0) to visualize the sub-network^29^. To understand the characteristic of the sub-network, we also calculated two network topological properties (degree and the shortest path) using the NetworkAnalyzer plugin in Cytoscape (V2.8.0).

## Additional Information

**How to cite this article**: Wei, R. *et al*. Concordance between somatic copy number loss and down-regulated expression: A pan-cancer study of cancer predisposition genes. *Sci. Rep.* **6**, 37358; doi: 10.1038/srep37358 (2016).

**Publisher's note:** Springer Nature remains neutral with regard to jurisdictional claims in published maps and institutional affiliations.

## Supplementary Material

Supplementary Information

Supplementary Table S1

Supplementary Table S2

Supplementary Table S3

Supplementary Table S4

Supplementary Table S5

Supplementary Table S6
