# Supplementary material for: Concordance between somatic copy number loss and down-regulated expression: A pan-cancer study of cancer predisposition genes
Source: Sci Rep. 2016 Dec 8;6:37358. doi: 10.1038/srep37358 (PMC5144096; doi:10.1038/srep37358)
Supplement: Supplementary Information [file srep37358-s1.doc]

**Concordance between somatic copy number loss and down-regulated expression: A** **pan-cancer study of cancer predisposition genes**

Ran Wei^1^, Ming Zhao^1^, Chun-Hou Zheng^1^, Min Zhao^2,*^, and Junfeng Xia^1,*^

^1^Institute of Health Sciences, School of Computer Science and Technology, Anhui University, Hefei, Anhui 230601, China

^2^School of Engineering, Faculty of Science, Health, Education and Engineering, University of Sunshine Coast, Maroochydore DC, Queensland, 4558, Australia

***Correspondence to:** Junfeng Xia, E-mail: jfxia@ahu.edu.cn

Min Zhao, E-mail: mzhao@usc.edu.au

**Supplementary Information**

**Supplementary Table S1**. The 128 human CPGs with frequent copy number loss (CNL).

**Supplementary Table S2**. The significantly enriched Gene Ontology (GO) annotations in the 128 human CPGs with frequent CNLs.

**Supplementary Table S3.** The frequency of GO term from 128 CPGs with frequent CNLs in randomly generated 128 CPGs with 100 permutations

**Supplementary Table S4.** The 49 CPGs with decreased gene expression induced by CNLs.

**Supplementary Table S5.** The CNV frequency of 49 CPGs with decreased gene expression induced by CNLs in pan-cancer.

**Supplementary Table S6.** The significantly enriched GO annotations in the 49 human CPGs with decreased gene expression induced by CNLs.
